# Presence of Belowground Neighbors Activates Defense Pathways at the Expense of Growth in Tobacco Plants

**DOI:** 10.3389/fpls.2019.00751

**Published:** 2019-06-11

**Authors:** Bin J. W. Chen, Roghieh Hajiboland, Sara Bahrami-Rad, Narges Moradtalab, Niels P. R. Anten

**Affiliations:** ^1^College of Biology and the Environment, Nanjing Forestry University, Nanjing, China; ^2^Department of Plant Science, University of Tabriz, Tabriz, Iran; ^3^Institute of Crop Science, University of Hohenheim, Stuttgart, Germany; ^4^Centre for Crop Systems Analysis, Wageningen University, Wageningen, Netherlands

**Keywords:** defense pathway, lignin, neighbor detection, *Nicotiana rustica*, phenolics, root interaction, self/non-self recognition

## Abstract

Plants can detect the presence of their neighbors belowground, often responding with changes in root growth for resource competition. Recent evidence also implies that perception of neighbors may also elicit defense responses, however, the associated metabolic activities are unclear. We investigated primary and defense-related secondary metabolisms and hormone expressions in tobaccos (*Nicotiana rustica*) grown either with own roots or roots of another conspecifics in hydroponic condition. The results showed that non-self root interaction significantly reduced photosynthetic activity and assimilate production, leading to a reduction of growth. Non-self interaction also modified plant phenylpropanoids metabolism, yielding higher lignin content (i.e., structural resistance) at whole plant level and higher phenolics accumulation (i.e., chemical defense) in roots. All these metabolic responses were associated with enhanced expressions of phytohormones, particularly jasmonic acid, salicylic acid and cytokinin in roots and abscisic acid in leaves, at the early stage of non-self interaction. Since the presence of neighbors often increase the probability of attacks from, e.g., pathogens and pests, this defense activation may act as an adaptation of plants to these possible upcoming attacks.

## Introduction

Plant roots can detect the presence of the roots of neighboring plants, and even identify the level of kin-ship of these neighbors, and respond accordingly (Chen et al., 2012; Dudley et al., 2013). Although the underlying mechanisms remain elusive, mounting evidence suggests that root-secreted chemicals act as the cues triggering the operation of genetically based neighbor detection (Biedrzycki et al., 2010; Semchenko et al., 2014; Kong et al., 2018; Yang et al., 2018). Even though neighbor presence can have a wide variety of effects on plants (Chen et al., 2014; Lankinen et al., 2016), studies so far reporting the responses of plants to the presence of belowground neighbors have mainly focused on the morphological aspects, such as root proliferation, biomass allocation and growth direction, that contribute to the competitive performance of plants in the impending competition for soil resources (Semchenko et al., 2014; Chen et al., 2015; Yang et al., 2018).

Because of their sessile living style, plants may be especially sensitive to cues that indicate environmental changes. For instance, they exhibit shade avoidance syndromes in response to a reduction of red/far-red ratio in light spectrum, which often indicates impending competition for light (Vandenbussche et al., 2005). Temperate trees can initiate autumn senescence in response to the shortening photoperiod, which in nature suggests the upcoming of cold winter (Fracheboud et al., 2009). The presence of neighbors can serve as cues that imply impending competitive circumstances in soils for plants. For example, when growing with *Festuca rubra*, *Plantago lanceolata* produces more roots than when growing with conspecific, and this response is likely associated with pre-emptive capture of resources (Padilla et al., 2013). In addition to indicating impending competition, the presence of neighbors may also convey other messages, including a higher probability of biotic attacks. Such neighbors may have been grown from seeds carrying pathogens and dispersed from elsewhere (Shade et al., 2017). Furthermore, the presence of neighbors also inevitably increases plant density, potentially increasing visibility to pests (Root, 1973) and increasing air humidity that favors development of some pathogens (Friesland and Schröedter, 1998). Therefore, it is reasonable to hypothesize that the presence of neighbors is a reliable warning signal for possible impending biotic attacks.

This could imply an overlap cross-talk between signaling pathways involved in the responses of plants to neighbors and to attackers. There is indeed some supporting evidence. A number of studies have demonstrated that many plant species upregulate the expressions of their defense response genes when growing with neighbors than when growing solitarily (Masclaux et al., 2012; Schmid et al., 2013; Bowsher et al., 2017; Markovic et al., 2019). The extent of such upregulations even positively correlates with the degree of genetic dissimilarity of the neighbors (Biedrzycki et al., 2011; Badri et al., 2012; Bowsher et al., 2017). However, besides these pieces of evidence from transcriptomic analyses, the subsequent regulation pathways and metabolic responses are still largely unknown. Among the limited evidence available, one study reported that when grown with conspecifics the species *Centaurea maculosa* enhanced its production of total phenolics (Broz et al., 2010), which are well-known as defense metabolites against stress and pathogen attacks (Lewis, 2017). This also implies that the perception of neighbors can induce modifications of phenylpropanoid pathways in plants.

An effective defense in plants often requires a substantial resource and energy investments which impose limitations on the growth and reproduction of plants (Neilson et al., 2013; Karasov et al., 2017). This entails that the expenditure for defense metabolism triggered by the presence of neighbors potentially can be at the expense of plant primary metabolism. Indeed, there is ample evidence showing that the expressions of photosynthesis related genes are downregulated in plants interacting with neighbors (Horvath et al., 2015; Moriles et al., 2017, but see Schmid et al., 2013). This can be further confirmed by some observations that plant biomass accumulation is often reduced in the presence of neighbors, even after controlling for constant nutrient supply per plant individual (Meier et al., 2013; Chen et al., 2015; Jacob et al., 2017, but see no difference in Markham and Halwas, 2011). However, direct evidence of neighbor-induced reduction in primary metabolic activities so far is still scarce.

Here we present the results of an experiment studying the consequences of root-mediated neighbor detection on primary metabolism and defense metabolism in plants of the model species tobacco (*Nicotiana rustica*) that were subjected either to self or to non-self root interaction in hydroponic condition. Measures of plant primary metabolism included the parameters of leaf gas exchange, root respiration, photosynthate and biomass production; and measures of plant defensive metabolism were the parameters in phenylpropanoid pathway. In addition, the levels of some important growth and defense-related phytohormones were also measured, due to their important regulatory roles in the metabolic pathways. We tested the hypothesis that tobacco plants respond to the presence of belowground neighbors with an activation of defense pathways at the expense of primary metabolism and growth.

## Materials and Methods

### Experimental Setup

Tobacco (*N. rustica* cv. Basmas) seeds were provided by the Agricultural and Natural Resources Research and Education Center, West Azarbaijan, Iran. Seeds were surface-sterilized using sodium hypochlorite containing 1% (w/v) active chlorine, and germinated on perlite in dark and moistened by distilled water. After the emergence of primary leaves, seedlings were transferred to light and irrigated with autoclaved (120°C for 30 min at 1.0 atm) half-strength modified Hoagland solution (Johnson et al., 1957). Thirty-day-old young seedlings with similar sizes (approximately 10 cm in height and three expanded leaves) were transferred into hydroponic pots filled with autoclaved full-strength modified Hoagland solution. Two days later, 1–2 cm of the apical region of the tap root of each plant was removed to trigger lateral root formation. Plants were further grown for 2 weeks to let them develop two lateral roots that were more or less equal in length.

**FIGURE 1:**
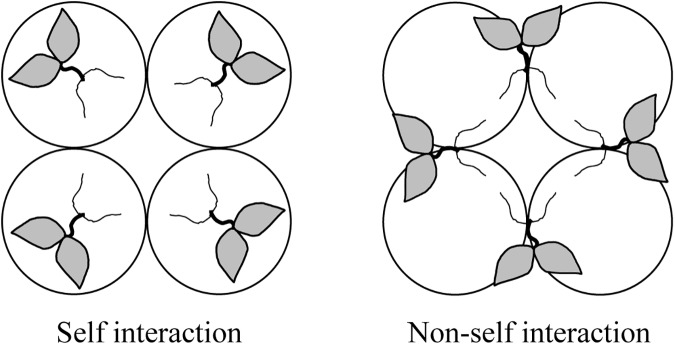
Schematic illustration of quadruple pots arrangement for the study of self/non-self root interactions in split-root tobacco plants. Circles stand for pots. One plant with all roots in one pot represents self interaction, while one plant with roots in two pots shared with other plants represent non-self interaction.

With a split-root design (Chen et al., 2015), similar-sized plants (approximately 15 cm in height and five expanded leaves) with two 10–12 cm long lateral roots were transferred into 1 L plastic hydroponic pots filled with aerated autoclaved full-strength modified Hoagland solution. Each pot contained either two roots from the same plant (self interaction) or from two different plants (non-self interaction) (Figure 1). Every four pots were glued together as a quadruple pot. There were 8 quadruple pots (i.e., 32 plants) for each root interaction treatment. Plants were grown in a growth chamber with a day/night temperature regime of 25/17°C, a relative humidity of 60%/70% and a photoperiod of 16/8 h at a photon flux density (PPFD) of about 300 μmol⋅m^-2^⋅s^-1^ provided by fluorescent lamps.

To minimize the competition for nutrients, the hydroponically based self/non-self root interaction treatments lasted for 2 weeks. In the experiment, plants were harvested in two batches, i.e., one after 1 week and the other after 2 weeks of treatments. In details, 32 plants belonging to eight quadruple pots of the two treatments were collected in each batch of harvest. After measuring leaf gas exchange parameters (as described below), plants were washed with double-distilled water, blotted, dried on filter papers and divided into shoots and roots. Some fresh subsamples were then taken for the determination of parameters of primary metabolism, root respiration, phenolics metabolism, phytohormones and biomass accumulation. The dry mass of plant leaves and roots were measured after 48 h oven-drying at the temperature of 70°C. In addition, mineral nutrient (including nitrogen, potassium, iron, and zinc) concentrations were also measured at the final harvest.

### Assay and Determinations of Primary and Defensive Metabolic Parameters

Gas exchange parameters were measured on attached leaves with a calibrated portable gas exchange system (LCA-4, ADC Bioscientific, United Kingdom) between 10:00 and 13:00 on fully expanded leaves under photosynthetically active radiation intensity at the leaf surface of 300–400 μmol⋅m^-2^⋅s^-1^. Reduction of triphenyltetrazolium chloride (TTC) (Sigma, United States) by tissue to the red-colored insoluble triphenylformazan (TF) that is analogous to the activity of the mitochondrial respiratory chain was measured in the fresh roots according to the method described by Ruf and Brunner (2003). In brief, fresh roots (100 mg) were sectioned into 1–2 mm segments and transferred to reaction tubes containing 2 mL of 100 mM potassium phosphate buffer (pH 7.0), 0.6% (w/v) TTC and 0.05% (v/v) Tween 20 (Sigma), and placed for 10 min under vacuum for infiltration. The samples were incubated for 20 h at 30°C, and then the TTC solution was decanted. For cell disruption and TF extraction, root segments were incubated with 1 mL of ethanol at 80°C for 15 min. Then, the reaction tubes were centrifuged at 10,000 rpm for 2 min. The absorbance of the supernatant was measured spectrophotometrically at the wavelength of 520 nm. The absorbance of the medium containing root samples, which were heat-inactivated by boiling in water at 100°C for 20 min before being incubated in TTC solution, served as controls. The root respiration rate was calculated as Δabs_520_ (between sample and control solutions) on the fresh weight base of roots.

For measuring non-structural carbohydrates, leaf and root samples were homogenized in 96% (v/v) ethanol at 4°C. After a centrifugation at 12,000 rpm for 15 min, supernatant was used for total soluble sugar analysis whereas the pellets were kept for starch analysis (Yemm and Willis, 1954). An aliquot of the supernatant was mixed with anthrone-sulfuric acid reagent and incubated for 10 min at 100°C. After cooling, the absorbance was spectrophotometrically determined at 625 nm. To determine starch concentration, the pellet was resuspended in a 4:1 (in volume) mixture of 8 N HCl and dimethylsulfoxide. Starch was dissolved for 30 min at 60°C under agitation. After a centrifugation at 12,000 rpm for 15 min, the supernatant was mixed with iodine-HCl solution. After 15 min at room temperature, the absorbance was spectrophotometrically determined at 600 nm.

To investigate the performance of phenylpropanoid pathway, phenylalanine ammonia lyase (PAL, EC 4.3.1.5) activity was determined for the leaves and roots. Fresh samples were extracted in 50 mM sodium borate buffer (pH 7.0) containing 2 mM EDTA, 18 mM 2-mercaptoethanol and 2% (w/v) insoluble polyvinylpyrrolidone. After a centrifugation at 12,000 rpm for 15 min, enzyme extract was mixed with the assay reagent containing 100 mM borate buffer (pH 8.8) and 12 mM L-phenylalanine, and incubated at 30°C. After 30 min reaction, the absorbance of trans-cinnamic acid was measured spectrophotometrically at 290 nm and its concentration was calculated with the coefficient of 9630 M^-1^⋅cm^-1^. The PAL activity was expressed as the conversion rate of L-phenylalanine to trans-cinnamic acid in mg^-1^ protein⋅min^-1^ (Dickerson et al., 1984). Polyphenol oxidase (PPO, EC 1.14.18.1) was extracted in 200 mM sodium phosphate buffer (pH 6.5), and its activity was assayed in a solution containing 10 mM pyrogallol and 200 mM sodium phosphate buffer (pH 6.5). The reaction was started by adding enzyme extract, and the oxidation of pyrogallol by enzyme activity was followed as the change in the absorbance at 334 nm for 10 min at 30°C. The activity was expressed as Δabs at 334 nm with a unit of mg^-1^ protein⋅min^-1^ (Singh et al., 1999). For the measure of soluble phenolics and cell wall lignin contents, fresh leaf and root tissues were extracted three times in 70% (v/v) aqueous methanol at 4°C in dark. After a centrifugation at 12,000 rpm for 15 min, the supernatant was used for phenolics measurement, while the pellet was used for isolation of cell walls (Solecka et al., 1999). The concentration of soluble phenolics in the supernatant was determined spectrophotometrically at 750 nm using Folin-Ciocalteu reagent and gallic acid as standard in the range between 0 to 0.5 mg⋅L^-1^ (Swain and Hillis, 1959). For isolation of cell walls, the pellet was freeze-dried and extracted sequentially with water (three times, each for 10 min), 2% (v/v) Triton in 1 M NaCl (three times, each for 10 min) and benzene-ethanol (1:1 in volume, for 1 h). After a centrifugation at 12,000 rpm for 15 min, the pellet (cell wall) was used for lignin measurement with the acetylbromide method (Morrison, 1972). In brief, 5 mg of air-dried cell wall preparation was treated with 2.4 mL freshly prepared acetyl bromide reagent (a mixture of acetylbromide and glacial acetic acid, 1:4 in volume) and 100 μL of 70% (v/v) HClO_4_, and heated at 70°C for 30 min with shaking at 5 min intervals. After cooling with ice, the digestion mixture was transferred to a 50 mL volumetric flask containing 10 mL of 2 M NaOH and 12 mL glacial acetic acid, and made up to 50 mL with glacial acetic acid. The lignin content was then determined by measuring the absorbance at the wavelength of 280 nm using a specific absorption coefficient of 20.0 L⋅g^-1^⋅cm^-1^.

For the determination of plant hormone concentrations, one gram of fresh leaf or root tissue was ground in the presence of liquid nitrogen and extracted twice with 2.5 mL of 80% (v/v) methanol. The samples were then further homogenized with an ultrasonicator (D-9 homogenizer, Miccra, Germany) for 75 s at 5,000 rpm. Then, 2 mL of the methanol extracts were transferred to microtubes and centrifuged at 6,000 rpm for 5 min. Thereafter, 350 μL of the supernatant was mixed with 700 μL ultra-pure water and centrifuged at 6,000 rpm for 5 min. The supernatant was filtered by membrane filter (Chromafil^®^ O-20/15 MS) to HPLC vials. The UHPLC-MS analyses were carried out on a Velos LTQ System (Thermo Fisher Scientific, United States) fitted with a Synergi Polar column 4 μm, 150 mm × 3.0 mm (Phenomenex, United States). The injection volume was 3 μL and the flow rate was adjusted to 0.5 mL⋅min^-1^. For gradient elution, water and two different concentrations (5% and 100%, v/v) of acetonitrile were used (Moradtalab et al., 2018). All standards were purchased from Sigma-Aldrich (United States) including (±)-jasmonic acid (JA), 3-indoleacetic-acid (IAA, the best known of auxins), gibberellic acid (GA), (±)-abscisic acid (ABA), trans-Zeatin (a member of cytokinins, CK), and salicylic acid (SA) that were used as external standards for quantitative analysis (Moradtalab et al., 2018).

To determine plant mineral nutrient concentrations, oven-dried leaf and root samples were wet-digested using perchloric acid for 2–3 h at the temperature around 250–300°C. The ash was dissolved in HCl and made up to a volume of 25 mL with distilled water. Concentration of nitrogen was determined using the indophenol blue method (Koroleff, 1983). Iron and zinc concentrations were determined by atomic absorption spectroscopy (AA6300, Shimadzu, Japan) and potassium was determined by flame photometry (PFP7, Jenway, United Kingdom).

### Statistical Analyses

In the analyses, a group of four plants from one quadruple-pot-set was regarded as one sample unit (or observation). Therefore, the experiment was a full factorial design with two factors (i.e., root interaction and harvest batch) and four replications. To examine the effects of root interaction (i.e., self vs. non-self interaction) and harvest batch (first vs. second week) on the growth (i.e., dry mass), and primary and defensive metabolic activities of tobacco plants, two-way ANOVAs (with root interaction, harvest batch and their interaction term as factors) followed by Tukey’s *post hoc* tests were performed. Since mineral nutrient concentrations were only measured at the final harvest, the effects of root interaction on them were tested using one-way ANOVAs. To fulfill the prerequisites (i.e., homoscedasticity and normality) of these parametric tests, some variables were log-transformed before the analyses (see details in Table 1 and Supplementary Table S1). When the prerequisites cannot be satisfied by data transformation, nonparametric tests (i.e., ANOVA with robust estimation) were performed. All the analyses were conducted in R ver. 3.5.3 (R Core Team, 2019). In addition to its basic system library, packages “car” (Fox and Weisberg, 2011) and “WRS2” (Mair and Wilcox, 2018) were, respectively, used for the Levene’s test for homogeneity of variance and ANOVA with robust estimation. The data and R scripts can be found in Supplementary Data Sheets S1, S2, respectively.

**Table 1:** Effects of root interaction (RI, i.e., self vs. non-self interaction), harvest batch (HB, i.e., first vs. second week) and their interaction term (RI×HB) on the measured parameters of tobacco plants.

Leaf (shoot) parameters	RI		HB		RI×HB		Root parameters	RI		BH		RI×HB	
	F	P	F	P	F	P		F	P	F	P	F	P
Dry mass	0.04	0.851	268.61	< 0.001	12.15	0.005	Dry mass^†^	10.42	0.007	131.76	< 0.001	2.54	0.137
Sugar	6.37	0.027	45.03	< 0.001	10.37	0.007	Sugar^‡^	26.10	0.001	19.87	0.002	9.92	0.013
Starch^†^	0.03	0.856	1315.35	< 0.001	6.90	0.022	Starch^†^	17.01	0.001	252.14	< 0.001	0.43	0.523
Protein	0.01	0.906	9.63	0.009	0.37	0.556	Protein	2.57	0.135	0.59	0.458	0.90	0.361
PAL	0.01	0.917	56.35	< 0.001	1.07	0.322	PAL	5.95	0.031	59.45	< 0.001	21.20	< 0.001
PPO^†^	8.03	0.015	18.93	< 0.001	0.35	0.564	PPO	17.32	0.001	3.55	0.084	0.37	0.553
Phenolics^‡^	8.20	0.013	1.39	0.261	0.05	0.822	Phenolics^†^	28.66	< 0.001	53.22	< 0.001	78.62	< 0.001
Lignin^†^	15.71	0.002	0.75	0.405	< 0.01	0.974	Lignin	28.99	< 0.001	2.55	0.136	< 0.001	0.998
IAA	1.11	0.313	31.27	< 0.001	0.15	0.703	IAA	2.56	0.136	3.21	0.098	1.52	0.241
GA	1.11	0.313	31.27	< 0.001	0.15	0.703	GA	2.56	0.136	3.21	0.098	1.52	0.241
CK^‡^	0.80	0.418	0.14	0.728	0.07	0.807	CK	12.37	0.004	10.92	0.006	3.78	0.076
ABA^‡^	8.56	0.028	5.58	0.061	6.13	0.053	ABA	0.85	0.374	0.35	0.567	0.90	0.361
JA^‡^	0.61	0.455	67.86	0.001	0.67	0.432	JA	25.89	< 0.001	48.31	< 0.001	10.46	0.008
SA^‡^	0.23	0.646	53.27	0.001	0.27	0.622	SA	25.89	< 0.001	48.31	< 0.001	10.46	0.008
Nitrogen	0.24	0.642					Nitrogen	3.18	0.125				
Potassium	5.79	0.053					Potassium	46.73	< 0.001				
Iron	0.04	0.854					Iron^‡^	0.07	0.801				
Zinc	0.89	0.381					Zinc	1.45	0.274				
NPR	13.05	0.004	0.01	0.912	0.38	0.551	RRR^‡^	128.34	0.001	551.84	0.001	97.78	0.001
SC	52.94	< 0.001	15.63	0.002	< 0.01	0.976							
TR	72.90	< 0.001	2.67	0.128	1.70	0.217							


*PAL, phenylalanine ammonia-lyase; PPO, polyphenol oxidase; JA, jasmonic acid; SA, salicylic acid; CK, cytokinins; ABA, abscisic acid; IAA, 3-indoleacetic-acid; GA, gibberellic acid; NPR, net photosynthetic rate; SC: stomatal conductance; TR, transpiration rate; RRR, root respiration rate. †indicates the variable was log-transformed before the parametric tests (i.e., ANOVAs); ‡indicates the parameter was analyzed using nonparametric tests (i.e., ANOVA with robust estimation).*

## Results

**FIGURE 2:**
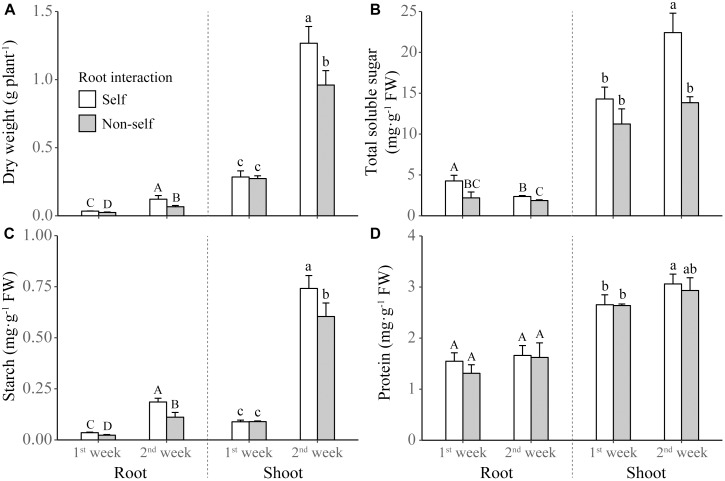
Dry weight **(A)** and concentrations of total soluble sugar **(B)**, starch **(C)**, and protein **(D)** in the shoot and roots of tobacco plants harvested at the end of first and second week of self and non-self interactions. Error bars denote 1 SD. Different uppercase (or lowercase) letters indicate significant differences between groups in roots (or shoot).

**FIGURE 3:**
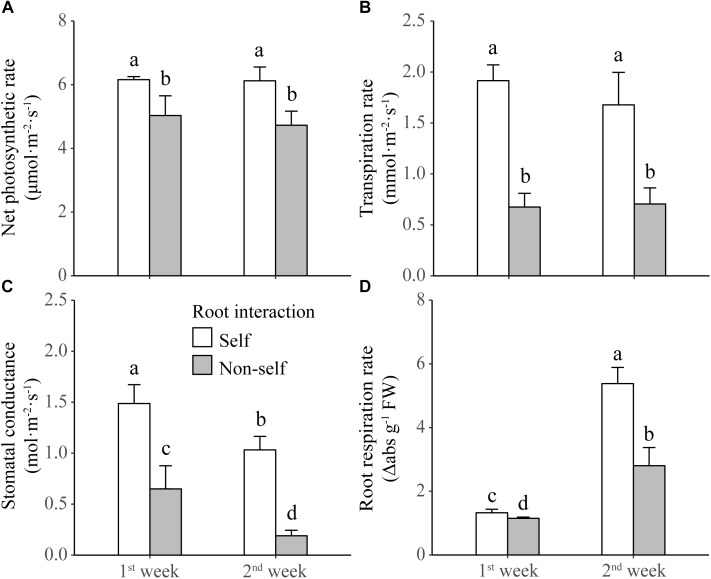
Net photosynthetic rate **(A)**, transpiration rate **(B)**, stomatal conductance **(C)**, and root respiration rate **(D)** of tobacco plants harvested at the end of first and second week of self and non-self interactions. Error bars denote 1 SD. Different letters indicate significant differences between groups.

Non-self-interacting plants (i.e., those interacting with roots of neighbors) produced significantly less shoot and root biomass than self-interacting plants (i.e., those interacted with own roots); and the extent of reductions became more profound after 2 weeks than after 1 week of non-self interaction (Table 1 and Figure 2A). Compared to self interaction, non-self interaction yielded lower soluble sugar and starch concentrations but had no effect on protein concentration in plants (Table 1 and Figure 2B–D). Significant reductions of sugar and starch concentrations occurred 1 week earlier in roots than in leaves (Figure 2B,C). Non-self interacting plants also had lower primary metabolic activities, in terms of lower net photosynthesis rates, transpiration rates, stomatal conductance and root respiration rates (Table 1 and Figure 3). The extent of reduction in root respiration rate became more profound after 2 weeks of non-self interaction (Table 1 and Figure 3D). Nutrient concentrations, as determined at the end of the experiment, generally remained at similar levels between non-self- and self-interacting plants, except for root potassium concentration which was higher in the former (Supplementary Figure S1).

**FIGURE 4:**
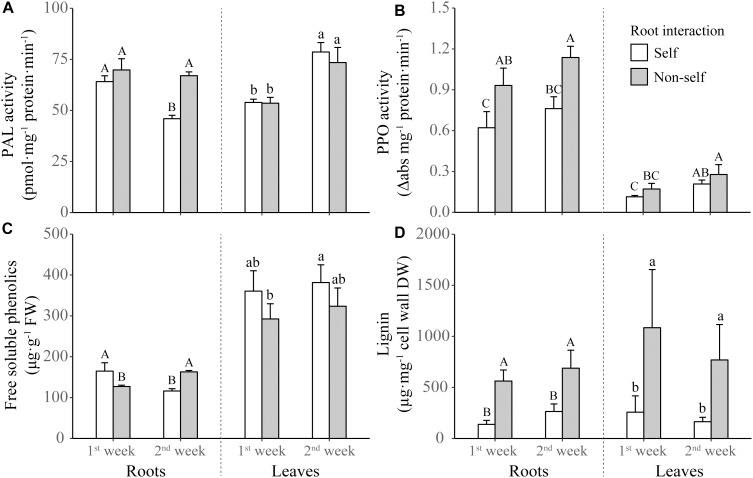
Activities of phenylalanine ammonia-lyase (PAL) **(A)** and polyphenol oxidase (PPO) **(B)**, and concentrations of free soluble phenolics **(C)** and lignin **(D)** in the leaves and roots of tobacco plants harvested at the end of first and second week of self and non-self interactions. Error bars denote 1 SD. Different uppercase (or lowercase) letters indicate significant differences between groups in roots (or leaves).

For the phenolics metabolizing enzymes and products, the activities of PAL and PPO in roots were elicited by non-self interaction (Table 1 and Figure 4A,B). However, the significantly increased activity in root PAL was only found after 2 weeks of non-self interaction (Figure 4A). Root interaction also significantly affected on the activity of leaf PPO (Table 1), which tended to be higher in non-self-interacting than self-interacting plants (Figure 4B). Although the concentration of root phenolics were higher in self-interacting plants in the first week of treatment, it became significantly higher in non-self-interacting plants after 2 weeks of treatment (Figure 4C). On the other hand, non-self-interacting plants consistently had significantly higher lignin contents in both leaves and roots than self-interacting plants throughout the 2 weeks (Figure 4D).

**FIGURE 5:**
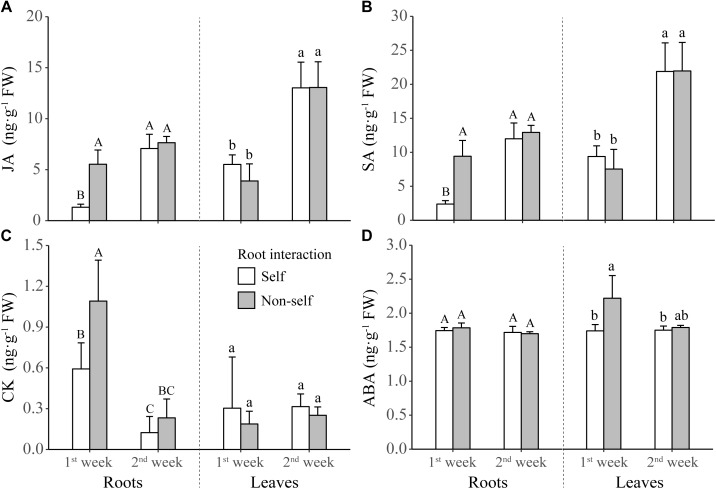
Concentrations of jasmonic acid (JA) **(A)**, salicylic acid (SA) **(B)**, cytokinin (CK) **(C)**, and abscisic acid (ABA) **(D)** in the leaves and roots of tobacco plants harvested at the end of first and second week of self and non-self interactions. Error bars denote 1 SD. Different uppercase (or lowercase) letters indicate significant differences between groups in roots (or leaves).

For the defense-related phytohormones, concentrations of JA and SA were significantly higher in the roots of non-self-interacting plants than those of self-interacting plants; however, these differences mainly occurred in the first week of interaction (Table 1 and Figure 5A,B). On the other hand, for the growth-related phytohormones, CK concentration in roots and ABA concentration in leaves were significantly higher in the first week of non-self interaction (Table 1 and Figure 5C,D); while the concentrations of IAA and GA remained similar between self- and non-self-interacting plants throughout the whole experiment (Table 1 and Supplementary Figure S2).

## Discussion

By setting up a self/non-self root interaction experiment, we found that our tobacco plants reduced primary carbon metabolic activities while enhanced defense-related phenolics metabolism when exposed to belowground neighbors. These metabolic responses were associated with enhanced expressions of phytohormones, particularly JA, SA, and CK in roots and ABA in leaves, at the early stage of non-self interaction and finally yielded a reduction in biomass production. These results hence support our hypothesis that in response to the presence of belowground neighbors plants activate their defense pathways at the expense of primary metabolism and growth. Below, we discuss the plausible mechanisms for the regulations of these neighbor-induced metabolic pathways.

Although, the underlying mechanisms for root-mediated neighbor detection and identity recognition remain elusive, mounting evidence suggests that the cues for perception are mostly related to chemicals, including proteins (Badri et al., 2012), secondary metabolites (Fang et al., 2013; Kong et al., 2018; Yang et al., 2018) and phytohormones (Kong et al., 2018), secreted as root exudates. Interestingly, a great portion of these chemicals also function as important metabolic regulators of plants in response to abiotic and biotic stress. For instance, cues for neighbor detection in wheat include JA and (-)-loliolide (Kong et al., 2018). The former is known as a ubiquitous phytohormone to elicit plant immunity against biotic attacks (Berens et al., 2017), and the latter also plays roles in regulating plant defense (Pan et al., 2009). Allantoin has been shown to be a cue for kin recognition in rice (Yang et al., 2018), and it also can enhance the production of ABA, subsequently activating JA signaling pathways (Takagi et al., 2016). Interestingly, we also observed enhanced expressions of defense-related phytohormones, especially JA, in non-self-interacting tobacco plants. However, our findings here only suggest that an upregulation of hormone metabolism in tobacco is induced by the presence of neighbors (also see reviews by Subrahmaniam et al., 2018 based on transcriptomic studies). To what extent these hormones also act as cues for neighbor detection in tobaccos is still unknown.

The upregulation of phytohormones likely accounted for the changes in the investigated metabolic activities in non-self-interacting tobaccos. For instance, JA and SA can adjust phenolics production via its regulations on the gene expression of lignin-synthesis-related enzymes, including PAL and PPO, in the phenylpropanoid pathway (Derksen et al., 2013). This may explain the enhanced activities of PAL and PPO in our study. The ABA mainly functions as a growth regulator and is involved in stress resilience by regulating stomatal conductance, i.e., an increase of ABA leads to stomatal closure in leaves (Munemasa et al., 2015). This may inevitably limit plant photosynthesis, thus echoing our observations of reduced photosynthetic activity and products. In addition, crosstalk between ABA and other defense phytohormones, such as JA and SA, can regulate the gene expressions in plant defense signaling (Derksen et al., 2013). The enhanced expression of another growth regulator CK (i.e., tZ here), as found in our study, can activate plant defense by inducing the expression of SA immune genes (Albrecht and Argueso, 2017). Prior increase in CK followed by wounding will induce higher levels of the JA precursor, leading to potential defense priming against herbivores (Naseem et al., 2014). Notably, these higher hormone expressions only occurred in the early stage (i.e., the first week) of interactions with non-self roots. Such an attenuation of hormonal signals in fact is quite common in biotic interactions in plants (Grant and Jones, 2009). For instance, a defense success of host plants often requires an attenuation of microbe-induced hormonal perturbations (Grant and Jones, 2009).

Our results clearly demonstrated that the phenolics metabolism is involved in the neighbor-induced defense responses. In response to the presence of neighbors, our tobacco plants consistently synthesized more lignin, which is predominantly deposited in cell walls during the secondary wall formation that reinforces the strength and rigidity of the walls and is a key component of response to stress (Le Gall et al., 2015). The process of lignification involves participations of PAL and PPO (O’Brien et al., 2012), thus may explain the increased activities of these particular enzymes measured in non-self-interacting tobaccos. These plants also tended to accumulate more soluble phenolics in roots. These compounds demonstrate a striking example of plasticity in metabolism enabling plants to cope with various conditions (Boudet, 2007). They play essential roles in mediating plant-microbe interactions, and many of them are classified as phytoalexins and synthesized in response to pathogen attack (Shalaby and Horwitz, 2015). When secreted into the rhizosphere, phenolics also benefit the mobilization and uptake of micronutrients, as well as the interactions with microorganisms (Dakora and Phillips, 2002). They can even function as allelotoxins suppressing neighbor’s growth (John and Sarada, 2012). This may also partially contribute to the growth reduction of non-self-interacting tobaccos.

In view of the above-mentioned activation of defense metabolic pathways, it is also not surprising that we found a cost, in terms of a reduction in primary metabolism, in tobacco plants exposed to non-self interaction. In fact, a downregulation of photosynthesis, including transcript levels of photosynthetic light reaction, pigment synthesis and carbon reduction cycle genes, in parallel with the upregulation of JA and SA, is commonly found in plants exposed to herbivore and pathogen attacks (Bilgin et al., 2010). Such responses allow plants to invest more resources in defense (Bolton, 2009). This could explain the reduction in plant biomass observed in our non-self-interacting tobaccos, and may also explain similar findings of neighbor-induced growth reduction in other studies (Meier et al., 2013; Chen et al., 2015; Jacob et al., 2017, but see no reduction in McNickle and Brown, 2014, and even overproduction of root mass in Gersani et al., 2001). No evidence of resource shortage in non-self-interacting plants (Supplementary Figure S1) further confirmed that such a reduction cannot be simply attributed to resource limitation in competition (also see discussions in Meier et al., 2013; Chen et al., 2015). Meanwhile, contrary to the increased root respiration in non-self-interacting pea plants reported by Meier et al. (2013), our tobacco plants showed a decline of root respiration. This discrepancy might be attributed to the fact that neighbor presence can elicit higher activities of alternative oxidase in roots of those peas (Meier et al., 2013), and that could not be detected by the TTC method used in our work.

Although our results are consistent with the hypothesized trade-off between defense and growth of plants, we still cannot exclude the possibility that the reduction in primary metabolism could be independent of neighbor-induced activation in plant defense metabolism. For example, an alternative explanation is that the growth reduction of non-self-interacting tobaccos may reflect a strategy utilized by them to avoid, or to lessen intraspecific competition. Therefore, further investigations are still needed. We are also aware that the setup of a strictly controlled and simplified system in our study may bring some extent of limitations on the implications of our results in the natural systems. For instance, root clipping as a control for the split-root treatment can simulate a mechanical stress from herbivore attacks. This may to some extent increase the expressions of defense related parameters in self-interacting plants. In addition, release rate of root exudates and their diffusion in the medium are likely to differ between hydroponic conditions and solid substrates (especially the soil) (Vranova et al., 2013). Therefore, the next important step is to verify whether non-self root interaction also can activate the defense of plants grown in soils.

To sum up, our study is among the first to demonstrate metabolic defense responses in plants exposed to the roots of neighbors. Such reactions might simply be the by-products of the cues utilized in the perception, since most cues accidently are also involved in the regulation of plant defense. Alternatively, these responses are adaptive, as the presence of neighbors could be associated with increased probability of biotic attacks. Cues indicating neighbor presence thus can be processed as warning signals for possible future attacks, and an activation of defense ahead of attacks would benefit the success of defense in plants. Therefore, this work expands the current knowledge of below-ground neighbor detection from the effects on resource competition to biotic defense, and provides a new scope of plant-plant root interactions. In addition to completely elucidate the metabolic pathways, more interesting questions can be raised. For example, how long will these neighbor-induced defense activities persist in the absence of attackers? Do the activities also involve more root secretion of defensive chemicals (e.g., phenolics) which may affect the rhizosphere microbiome and thus plant-microbe interactions in soil? And to what extent is the upregulation of defense response depend on the kin-ship (e.g., intra- vs. inter-cultivar; intra- vs. interspecific) between interacting plants?

## Author Contributions

RH designed the research. SB-R and NM conducted the experiments. BC and RH analyzed the data. BC, RH, and NA wrote the manuscript. All authors read and approved the manuscript.

## Conflict of Interest Statement

The authors declare that the research was conducted in the absence of any commercial or financial relationships that could be construed as a potential conflict of interest.

## Supplementary Material

FIGURE S1 Concentrations of mineral nutrients, i.e., nitrogen, potassium, iron and zinc, in the leaves and roots of tobacco plants at the end of first and second week of self and non-self root interactions.

FIGURE S2 Concentrations of gibberellic acid (GA) and 3-indoleacetic-acid (IAA) in the leaves and roots of tobacco plants harvested at the end of first and second week of self and non-self root interactions.

TABLE S1 Results of the tests for normal distribution of residuals and homogeneity of variation.

DATA SHEET S1 Original data collected from the experiment.

DATA SHEET S2 R scripts used in the analyses.
