# Supplementary material for: Presence of Belowground Neighbors Activates Defense Pathways at the Expense of Growth in Tobacco Plants
Source: Front Plant Sci. 2019 Jun 11;10:751. doi: 10.3389/fpls.2019.00751 (PMC6584819; doi:10.3389/fpls.2019.00751)
Supplement: FIGURE S1 — Concentrations of mineral nutrients, i.e., nitrogen, potassium, iron and zinc, in the leaves and roots of tobacco plants at the end of first and second week of self and non-self root interactions. [file Table_1.DOCX]

Supplementary Material

**Supplementary Table S1** Results of the tests for normal distribution of residuals (using Shapiro-Wilk normality test) and homogeneity of variation (using Levene's test)

| Leaf (shoot) parameter | Shapiro-Wilk test | |  | levene's Test | |  | Root parameter | Shapiro-Wilk test | |  | levene's Test | |
| --- | --- | --- | --- | --- | --- | --- | --- | --- | --- | --- | --- | --- |
|  | *w* | *P* |  | *F* | *P* |  |  | *w* | *P* |  | *F* | *P* |
| Dry mass | 0.920 | 0.169 |  | 1.414 | 0.287 |  | Dry mass† | 0.975 | 0.917 |  | 2.436 | 0.115 |
| Sugar | 0.918 | 0.158 |  | 0.728 | 0.555 |  | Sugar‡ | 0.893 | 0.062 |  | 7.423 | 0.005 |
| Starch† | 0.932 | 0.265 |  | 0.349 | 0.791 |  | Starch† | 0.963 | 0.714 |  | 0.752 | 0.542 |
| Protein | 0.967 | 0.785 |  | 0.904 | 0.468 |  | Protein | 0.951 | 0.508 |  | 0.403 | 0.753 |
| PAL | 0.963 | 0.709 |  | 1.359 | 0.302 |  | PAL | 0.947 | 0.440 |  | 0.891 | 0.474 |
| PPO† | 0.980 | 0.964 |  | 1.709 | 0.218 |  | PPO | 0.937 | 0.310 |  | 0.380 | 0.769 |
| Phenolics‡ | 0.878 | 0.036 |  | 0.073 | 0.974 |  | Phenolics† | 0.893 | 0.063 |  | 2.642 | 0.097 |
| Lignin† | 0.964 | 0.740 |  | 1.748 | 0.210 |  | Lignin | 0.936 | 0.299 |  | 2.102 | 0.153 |
| IAA | 0.974 | 0.904 |  | 1.517 | 0.260 |  | IAA | 0.949 | 0.472 |  | 0.724 | 0.557 |
| GA | 0.974 | 0.904 |  | 1.517 | 0.260 |  | GA | 0.949 | 0.472 |  | 0.724 | 0.557 |
| CK‡ | 0.838 | 0.009 |  | 1.128 | 0.377 |  | CK | 0.921 | 0.177 |  | 0.412 | 0.748 |
| ABA‡ | 0.830 | 0.012 |  | 1.225 | 0.351 |  | ABA | 0.947 | 0.450 |  | 1.134 | 0.375 |
| JA‡ | 0.858 | 0.018 |  | 0.346 | 0.793 |  | JA | 0.984 | 0.991 |  | 1.196 | 0.356 |
| SA‡ | 0.824 | 0.010 |  | 0.290 | 0.831 |  | SA | 0.984 | 0.991 |  | 1.196 | 0.356 |
| Nitrogen | 0.946 | 0.674 |  | 0.014 | 0.909 |  | Nitrogen | 0.962 | 0.825 |  | 0.756 | 0.418 |
| Potassium | 0.948 | 0.696 |  | 0.081 | 0.786 |  | Potassium | 0.983 | 0.977 |  | 0.272 | 0.621 |
| Iron | 0.873 | 0.162 |  | 1.171 | 0.321 |  | Iron‡ | 0.924 | 0.462 |  | 7.217 | 0.036 |
| Zinc | 0.915 | 0.388 |  | 0.060 | 0.815 |  | Zinc | 0.904 | 0.312 |  | 0.431 | 0.536 |
| NPR | 0.952 | 0.521 |  | 0.416 | 0.745 |  | RRR‡ | 0.955 | 0.578 |  | 34.055 | <0.001 |
| SC | 0.930 | 0.240 |  | 1.854 | 0.191 |  |  |  |  |  |  |  |
| TR | 0.968 | 0.813 |  | 1.164 | 0.364 |  |  |  |  |  |  |  |

PAL, phenylalanine ammonia-lyase; PPO, polyphenol oxidase; JA, jasmonic acid; SA, salicylic acid; CK, cytokinins; ABA, abscisic acid; IAA, 3-indoleacetic-acid; GA, gibberellic acid; NPR, net photosynthetic rate; SC: stomatal conductance; TR, transpiration rate; RRR, root respiration rate. † indicates the variable was log-transformed before the parametric tests (i.e. ANOVAs); ‡ indicates the parameter was analyzed using nonparametric tests (i.e. ANOVA with robust estimation).

**Supplementary Figures**


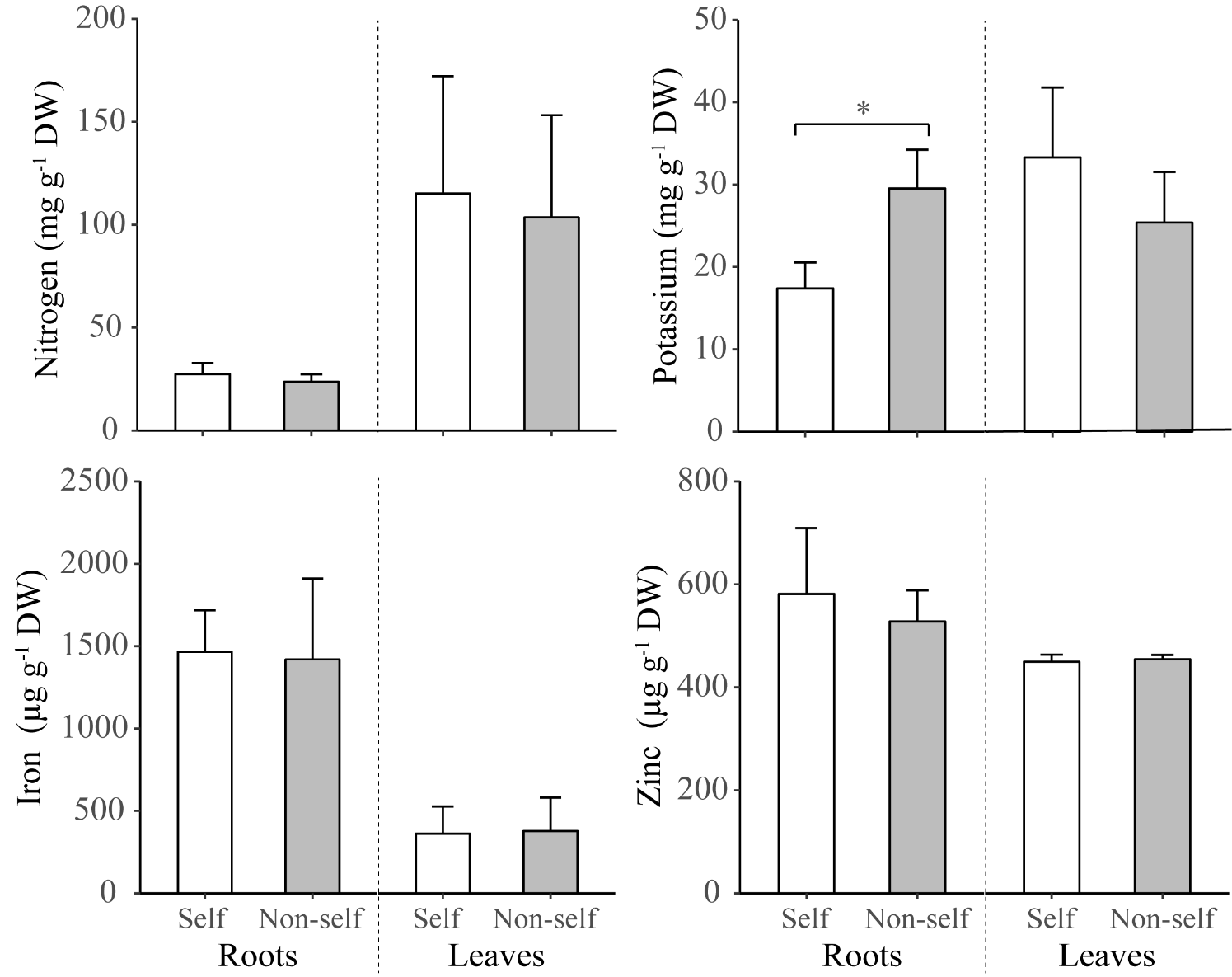


**Figure S1** Concentrations of mineral nutrients, i.e. nitrogen, potassium, iron and zinc, in the leaves and roots of tobacco plants at the end of first and second week of self and non-self root interactions. Error bars denote 1 *SD*. * indicates a significant difference between groups.


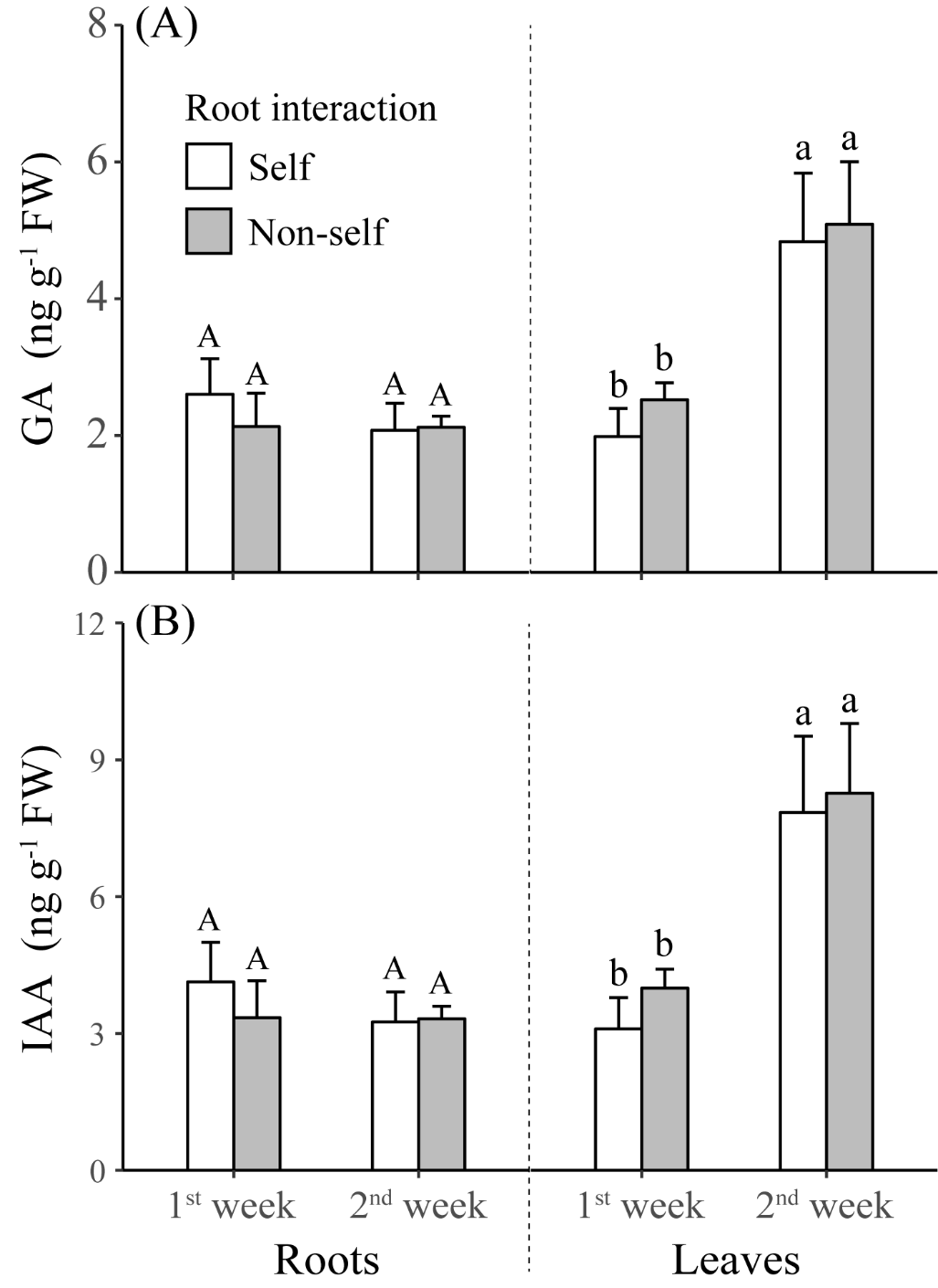


**Figure S2** Concentrations of (A) gibberellic acid (GA) and (B) 3-indoleacetic-acid (IAA) in the leaves and roots of tobacco plants harvested at the end of first and second week of self and non-self root interactions. Error bars denote 1 *SD*. Different uppercase (or lowercase) letters indicate significant differences between groups in roots (or leaves).
