# Supplementary material for: Presence of Belowground Neighbors Activates Defense Pathways at the Expense of Growth in Tobacco Plants
Source: Front Plant Sci. 2019 Jun 11;10:751. doi: 10.3389/fpls.2019.00751 (PMC6584819; doi:10.3389/fpls.2019.00751)
Supplement: DATA SHEET S2 — R scripts used in the analyses. [file Data_Sheet_2.pdf]

```
setwd("...")    # set your own work direction
dat0 <- read.csv("Appendix1.csv")    # input the csv data
head(dat0)    # have a glance of the data structure

# install.packages("car")    # run this, if package "car" has not been installed yet
require(car)    # for the Levene's test for homogeneity of variance

# install.packages("WRS2")    # run this, if package "WRS2" has not been installed yet
require(WRS2)    # for ANOVA with robust estimation

# column numbers of parameters in the dataset "dat0":
# mass (5); sugar (6); starch (7); protein (8); NPR (9); TR (10); SC (11); RRR (12); PAL (13);
# PPO (14); phenolics (15); lignin (16); IAA (17); GA (18); CK (19); ABA (20); JA (21); SA (22)
dat1 <- dat0[which(dat0$organ == "root"), c(1:3, 7)]    # create a sub-dataset for the analyses
# here, "root" (or "leaf") indicates that a root (or leaf) parameter is selected; and the number
# "7" indicates that the parameter in the 7th column (starch) of the full dataset is selected

dat1$value = dat1[,4]    # for convenience, copy the values of the selected parameter (in the
                         # 4th column of the sub-dataset) to a new variable named "value"
dat1$lnvalue = log(dat1$value)    # log-transform the values of variable "value" and
                                  # copy them to a new variable named "lnvalue"

# analysis for the selected parameter with original values
mod1 <- lm(value ~ neighbour*harvest, data = dat1)
shapiro.test(residuals(mod1))    # shapiro test for the normal distribution of residuals
leveneTest(value ~ neighbour*harvest, data = dat1)    # Levene's test
Anova(mod1, type = 3)     # type III based ANOVA
TukeyHSD(aov(value ~ neighbour*harvest, data = dat1))    # Tukey's post-hoc test

# if homoscedasticity or normality is not satisfied, log-transformed parameter values are used
mod1.log <- lm(lnvalue ~ neighbour*harvest, data = dat1)
shapiro.test(residuals(mod1.log))
leveneTest(lnvalue ~ neighbour*harvest, data = dat1)
Anova(mod1.log, type = 3)
TukeyHSD(aov(lnvalue ~ neighbour*harvest, data = dat1))

# If data transformation does not work, nonparametric test (two-way ANOVA with robust
# estimation) is performed.
t2way(value ~ neighbour*harvest, data = dat1)
mcp2atm(value ~ neighbour*harvest, data = dat1)
```

```
# column numbers of parameters in the dataset "dat0":
# N (23), K (24), Fe (25), Zn (26)
dat2 <- na.omit(dat0[which(dat0$organ == "root"), c(1:3, 23)])
# here, the number "23" indicates that the parameter in the 23rd column (N) of the full dataset
# is selected

dat2$value = dat2[,4]
dat2$lnvalue = log(dat2$value)

# analysis for the parameter with original values
mod2 <- lm(value ~ neighbour, data = dat2)
shapiro.test(residuals(mod2))
leveneTest(value ~ neighbour, data = dat2)
Anova(mod2, type = 3)

# if homoscedasticity or normality is not satisfied, parameter values are log-transformed
mod2.log <- lm(lnvalue ~ neighbour, data = dat2)
shapiro.test(residuals(mod2.log))
leveneTest(lnvalue ~ neighbour, data = dat2)
Anova(mod2.log, type = 3)

# If data transformation does not work, nonparametric test (one-way ANOVA with robust
# estimation) is performed.
t1way(value ~ neighbour, data = dat2)
```
